# Genetic and hemostatic characterization of PAI-1 deficiency and isolated hyperfibrinolysis: data from the RBiN study

**DOI:** 10.1016/j.rpth.2026.106928

**Published:** 2026-08-27

**Authors:** Bauke Haisma, Thomas Nieuwenstein, Sanna R. Rijpma, Annet Simons, Nicole M.A. Blijlevens, Waander L. van Heerde, Saskia E.M. Schols

**Affiliations:** 1 Department of Hematology, Radboud University Medical Center, Nijmegen, the Netherlands; 2 Department of Hematologie, Radboud University Medical Center, Hemophilia Treatment Center Nijmegen-Eindhoven-Maastricht, Nijmegen, the Netherlands; 3 Department of Laboratory Medicine, Laboratory of Hematology, Radboud University Medical Center, Nijmegen, the Netherlands; 4 Department of Human Genetics, Radboud University Medical Center, Nijmegen, the Netherlands; 5 Enzyre BV, Noviotech Campus, Nijmegen, the Netherlands

**Keywords:** exome, exome sequencing, genetic polymorphism, genetic promoter region, genotype, inherited blood coagulation disorders, mutation, plasminogen activator inhibitor-1 deficiency

## Abstract

**Background:**

Plasminogen activator inhibitor 1 (PAI-1) is a key regulator of fibrinolysis, and its deficiency causes bleeding symptoms. PAI-1 deficiency is rare, and pathogenic *SERPINE1* variants are only sporadically reported.

**Objectives:**

To assess *SERPINE1* variant occurrence and plasmin generation in patients with PAI-1 deficiency compared to patients with isolated hyperfibrinolysis or rare coagulation factor deficiencies (RCFDs).

**Methods:**

This substudy of the nationwide, cross-sectional RBiN cohort included patients with PAI-1 deficiency, isolated hyperfibrinolysis, and RCFDs. *SERPINE1* variants were assessed by targeted exome sequencing and promoter polymorphisms by polymerase chain reaction and Sanger sequencing. Plasmin generation was measured using the Nijmegen Hemostasis Assay.

**Results:**

Fourteen patients with PAI-1 deficiency, 15 with isolated hyperfibrinolysis, and 214 with RCFDs were included. *SERPINE1* variants were identified in one patient with PAI-1 deficiency, one with isolated hyperfibrinolysis, and 30 with RCFDs. Variants p.Ala15Thr (rs6092) and p.Val17Ile (rs6090) were most frequently observed and did not significantly affect PAI-1 antigen levels (median 7.8 and 9.0 vs 7.3 ng/mL without variant) or bleeding scores (8 and 12.5 vs 10). The *SERPINE1* promoter polymorphisms c.-675 4G/5G (rs1799889) and c.-844A>G (rs2227631) were not associated with PAI-1 antigen levels or bleeding severity. Patients with PAI-1 deficiency or isolated hyperfibrinolysis showed significantly shorter fibrin lysis times (median 74% and 66%) and higher plasmin peak heights (145% and 170%) than healthy controls (100%).

**Conclusion:**

PAI-1 deficiency and isolated hyperfibrinolysis showed enhanced plasmin generation, but were not linked to *SERPINE1* variants or promoter polymorphisms. Further studies should explore noncoding, regulatory, and epigenetic mechanisms underlying PAI-1 deficiency.

## Essentials

• PAI-1 deficiency is a rare bleeding disorder with a distinct bleeding phenotype.
• Pathogenic SERPINE1 variants have only sporadically been reported in patients with PAI-1 deficiency.
• Patients with PAI-1 deficiency or isolated hyperfibrinolysis in the RBiN study showed a distinctly increased plasmin generation profile.
• Future genotyping of PAI-1 deficiency should entail exploration of non-coding, regulatory and epigenetic mechanisms.

## 1 Introduction

Plasminogen activator inhibitor type 1 (PAI-1) is a serine protease inhibitor and a key regulator of the fibrinolytic pathway, downregulating fibrinolysis through direct, noncompetitive inhibition of tissue-type plasminogen activator and urokinase plasminogen activator [1,2]. PAI-1 deficiency results in enhanced fibrinolysis and a moderate bleeding phenotype, mainly characterized by menorrhagia, (delayed) bleeding after trauma, surgery or tooth extraction, ecchymoses, and epistaxis [1,[3], [4], [5], [6]]. Severe bleeding manifestations, including intracerebral bleeding and hemarthrosis, are reported in a minority of patients [[6], [7], [8], [9], [10]].

PAI-1 deficiency has been described only in case reports and small case series, and its true prevalence is unknown, suggesting that it is an extremely rare disorder [1,[3], [4], [5], [6], [7], [8], [9], [10]]. However, the true prevalence of PAI-1 deficiency may be underestimated. Routine coagulation assays are typically normal in affected individuals, and many specialized laboratories do not have access to PAI-1 antigen and activity assays [7]. PAI-1 measurements require specific assays, and levels show pronounced diurnal variation, necessitating morning sampling after an overnight fasting period [11]. Limitations of current activity assays further complicate diagnosis, as levels below the lower limit of detection may still fall within the normal reference range [4,12]. Therefore, dysproteinemic forms of PAI-1 deficiency (reduced activity despite normal antigen levels) cannot be reliably identified with current activity assays, and PAI-1 antigen measurement remains essential for diagnosis [1,12,13].

PAI-1 is encoded by the *SERPINE1* gene on chromosome 7q22. Pathogenic *SERPINE1* variants have been identified in only a limited number of families with PAI-1 deficiency [6,[14], [15], [16], [17], [18]]. Many individuals with biochemical evidence of PAI-1 deficiency do not carry coding variants in *SERPINE1*, and it remains unclear whether other currently unrecognized variants or regulatory mechanisms, including promoter polymorphisms, may contribute to reduced PAI-1 levels. This study aims to investigate whether genetic variants in the *SERPINE1* gene can be identified in patients with an established biochemical PAI-1 deficiency, and whether these variants correlate with PAI-1 antigen levels or bleeding phenotype. For comparison, we also examined patients with isolated hyperfibrinolysis or rare coagulation factor deficiencies (RCFDs) from the same clinical study. A secondary objective was to explore the association of the -675 4G/5G (c.-675 4G/5G, rs1799889) and -844 A>G (c.-844 A>G, rs2227631) promoter polymorphisms with PAI-1 antigen levels and bleeding phenotype. Finally, we aimed to assess whether patients with PAI-1 deficiency or isolated hyperfibrinolysis exhibit enhanced plasmin generation, and whether this correlates with PAI-1 antigen levels, bleeding phenotype and/or the presence of *SERPINE1* variants or promoter polymorphisms.

## 2 Methods

### 2.1 The RBiN study

This substudy is part of the nationwide cross-sectional Rare Bleeding disorders in the Netherlands (RBiN) study, which included patients from all 6 Dutch Hemophilia Treatment Centers (HTCs). Participants were eligible if they had been diagnosed with a rare coagulation factor deficiency or a fibrinolytic disorder after referral to an HTC for a bleeding tendency, a positive family history of a rare bleeding disorder, or abnormal laboratory results from prior testing. Individuals aged one year or older were enrolled between October 2017 and November 2019. Further details on the study design and inclusion criteria have been published previously [19]. In total, 263 patients participated. Ethical approval was obtained from the Medical Ethics Committee of Arnhem-Nijmegen (study number NL61027.091.17), and written informed consent was provided by all participants or their legal guardians.

### 2.2 Patient selection

For the current analysis, we selected participants with PAI-1 deficiency or isolated hyperfibrinolysis. PAI-1 deficiency was defined as undetectable PAI-1 activity (<1.0 ng/mL) together with antigen levels below 3.4 ng/mL, corresponding to the 2.5th percentile of the reference population established in 120 healthy individuals (reference range, 3.4-39 ng/mL, defined as the 2.5th to 97.5th percentile). Isolated hyperfibrinolysis was determined in patients without PAI-1 or α2-antiplasmin deficiency, but with evidence of hyperfibrinolysis, defined as a euglobulin clot lysis time ratio ≥5.8 before and after tourniquet application, based on a locally validated assay (reference range, 1.2-5.7) [1,19]. Reference ranges were previously established using samples from 120 healthy individuals (68/120 women [57%], median age 46 years, interquartile range [IQR], 38-55) in accordance with current guidelines. The 2 hyperfibrinolytic groups were compared with RBiN study participants diagnosed with a deficiency of fibrinogen, coagulation factor (F) FII, FV, FVII, FX, FXI, or FXIII, or with combined FV and FVIII deficiency; this group will hereafter be referred to as patients with RCFDs. Fibrinogen disorders were defined as a persistently reduced fibrinogen activity below 1800 mg/L in the absence of an acquired cause, with a concordantly reduced antigen classified as afibrinogenemia or hypofibrinogenemia and a disproportionately elevated antigen relative to activity classified as dysfibrinogenemia. Deficiencies of other coagulation factors were defined as factor activity below the following thresholds, also in the absence of an acquired cause: FII <70%, FV <60%, FV and FVIII <60% for both factors, FVII <60%, FX <60%, FXI <70%, and FXIII <70%.

### 2.3 Study visit, bleeding phenotype, and laboratory assessment

During the single study visit, data on age, sex, body mass index, and bleeding severity were collected. Bleeding scores were assessed using the International Society on Thrombosis and Haemostasis Bleeding Assessment Tool (ISTH-BAT) by a single investigator. Blood samples were obtained via venipuncture, with a dummy tube drawn first, followed by a BD Vacutainer 3.2 mL citrate tube containing 0.109 M sodium citrate (Becton Dickinson, cat# 363080), filled to the indicated volume on the label. Given the susceptibility of PAI-1 levels to preanalytical and physiological variation, patients were instructed to refrain from tranexamic acid use for 3 days before the visit, to avoid alcohol consumption on the preceding day, and to consume only water and a light, low-fat breakfast on the morning of the visit; blood was drawn between 08:30 and 09:30 after a regular sleep–wake cycle. Tubes were promptly centrifuged at 4200 × *g* for 15 minutes at room temperature (20°C-25°C), and plasma was flash-frozen on-site at −80°C to preserve sample integrity. All laboratory analyses were conducted at the hematology laboratory of the Radboud University Medical Center to ensure uniformity and precision. PAI-1 antigen and activity were measured using ZYMUTEST PAI-1 antigen and activity kits (Hyphen BioMed; cat# RK012A and RK019A, respectively). Both assays were validated in our laboratory before use; assay performance characteristics are provided in Supplementary Table 3.

### 2.4 Genetic analysis

Targeted exome sequencing was conducted at the Human Genetics Department of the Radboud University Medical Center, using genomic DNA extracted from EDTA blood. Exonic enrichment and library preparation were performed using the Twist Human Core Exome Kit, followed by high-throughput sequencing on Illumina NovaSeq 6000 and/or HiSeq platforms. The obtained sequences were aligned to the hg19 reference genome using the Burrows-Wheeler Aligner, and variant calling was performed using the Genome Analysis Toolkit, with a minimum exon coverage requirement of >40× [20,21]. Variant annotation was performed using a custom in-house diagnostic procedure. Variants were filtered using a targeted gene panel consisting of 156 genes associated with thrombotic and hemostatic disorders (Supplementary Table 1) [[22], [23], [24]]. Copy number variants were detected through the copy number inference from exome reads pipeline [25]. The evaluation of variants was independently performed by 2 clinical laboratory geneticists (A.S. and J.W.), taking into account previous research, available databases (such as GnomAD v2.1.1), functional assay results, and predictive tools, including Alamut Visual Plus (version 1.6.1; SOPHiA GENETICS) [22]. Variants were classified in accordance with the guidelines of the American College of Medical Genetics and Genomics and the Association for Molecular Pathology [26]. For the *SERPINE1* gene, all nonsynonymous exonic (ie, coding) variants were assessed, whereas for the remaining genes in the panel, analysis was restricted to variants of uncertain significance, likely pathogenic, and pathogenic variants (ACMG classes, 3-5). For targeted analysis of the most common *SERPINE1* promoter polymorphisms, additional polymerase chain reaction (PCR) was performed in participants with PAI-1 deficiency or isolated hyperfibrinolysis, focusing on the -844 A>G and -675 4G/5G polymorphisms. In short, a PCR was performed on genomic DNA to enrich for the region of -1596 to -217 to the transcription start site of *SERPINE1* (Supplementary Table 2). PCR products were processed using the NucleoSpin Gel & PCR Clean-up kit (Macherey Nagel, cat#740609.250). The resulting product was used to perform Sanger sequencing on the region of -1036 to -441 to transcription start site of *SERPINE1*. Presence and zygosity of -844 A>G and -675 4G/5G polymorphisms were manually assessed.

### 2.5 Plasmin generation assay

Plasmin generation was assessed using the Nijmegen Hemostasis Assay [27]. Citrated plasma was mixed with low-concentration tissue factor (0.28 pM), recombinant tissue-plasminogen activator (190 IU/mL), cephalin as a phospholipid source, and a plasmin-specific fluorescent substrate. The reaction was initiated by adding calcium (final concentration of 16.7 mM), and fluorescence was recorded at excitation/emission wavelengths of 485/520 nm. Plasmin generation was calibrated using calibration curves prepared with human plasmin, and each run included normal pooled plasma from 60 healthy donors as a control. Proteolytic activity of plasmin was assessed from the first derivative of the fluorescence signal, with analysis focused on fibrin lysis time, plasmin peak height, and plasmin velocity index (Supplementary Figure 1) [27,28]. Parameters were normalized to the mean values from 37 healthy individuals (median age, 46 years; 57% female). Plasmin generation was measured in patients with PAI-1 deficiency and isolated hyperfibrinolysis, as well as in RCFD patients from the RBiN study with deficiencies in FII, FV, FV/FVIII, FVII, FX, and FXI. The RCFD patients served as a control group, representing individuals with bleeding disorders but without primary hyperfibrinolysis. Moreover, this approach allowed us to assess whether reduced thrombin generation in the latter group would affect plasmin generation.

### 2.6 Statistical analysis

Continuous variables are reported as medians with IQRs, and categorical variables as counts and percentages. Differences in categorical variables were assessed using Fisher’s exact test, while differences in continuous variables were evaluated with 2-sided anova followed by post-hoc pairwise *t*-tests. *P* values were adjusted for multiple comparisons using the Bonferroni method, and *P* values < .05 divided by the number of comparisons were considered statistically significant. All figures were created using R version 4.2.2 (R Foundation for Statistical Computing, Vienna, Austria).

## 3 Results

### 3.1 Patient characteristics

A total of 14 patients with PAI-1 deficiency, 15 patients with isolated hyperfibrinolysis, and 214 patients with RCFDs were included in the RBiN study (Table 1). The latter group consisted of 42 patients with a-, hypo-, or dysfibrinogenemia and 16, 27, 6, 58, 8, 43, and 14 patients with FII, FV, combined FV and FVIII, FVII, FX, FXI, and FXIII deficiency, respectively (Supplementary Table 4), of whom 94% had documented bleeding symptoms and 80% had a formally elevated ISTH-BAT score. In this group, 7% of patients had PAI-1 antigen levels below the 5th percentile, which is consistent with the expected 5%. In contrast, by definition, all patients with PAI-1 deficiency had antigen levels below the 2.5th percentile of the reference population. As expected, PAI-1 antigen levels were significantly lower in the PAI-1 deficiency group (*P* < .01), while euglobin clot lysis time ratios were significantly higher in the isolated hyperfibrinolysis group. Compared with the RCFD group, the proportion of female participants, median age, and ISTH-BAT score were higher in both the PAI-1 deficiency and isolated hyperfibrinolysis groups; however, except for sex, these differences did not reach statistical significance. Other characteristics, including body mass index and α2-antiplasmin concentration, were comparable across groups. In patients with PAI-1 deficiency or isolated hyperfibrinolysis, no concurrent bleeding disorders—such as von Willebrand disease, coagulation factor deficiencies, or α2-antiplasmin deficiency—were identified. All patients in these 2 groups had a documented bleeding tendency and an elevated ISTH-BAT score at the time of inclusion; individual patient data, including predominant bleeding manifestations, are provided in Supplementary Table 5.

**Table 1:** Characteristics of studied patients with PAI-1 deficiency, isolated hyperfibrinolysis, or rare coagulation factor deficiencies.

Characteristics	PAI-1 deficiency	Isolated hyperfibrinolysis	Rare coagulation factor deficiencies
**Number of patients(n)**	14	15	214
**Proportion of female patients(%)**	14 (100)	13 (86)	124 (58)
**Age (y)**	45 (36-52)	52 (37-65)	36 (18-56)
**Body mass index (kg/m^2^)**	25.2 (22.4-27.5)	24.7 (21.6-26.2)	23.7 (20.4-27.3)
**ISTH-BAT score**	11.5 (10-19.5)	12 (10.0-14.5)	8 (4-14)
**PAI-1 antigen (ng/mL)**	2.5 (1.9-2.7)	6.0 (4.3-7.8)	8.0 (4.5-14.8)
**Euglobin clot lysis time ratio**	3.8 (2.6-4.8)	9.0 (6.5-9.9)	Not measured
**Tissue plasminogen activator (IU/mL)**	Not measured	6.7 (4.7-27)	Not measured
**α-2-antiplasmin (%)**	115 (102-119)	102 (98-109)	108 (100-117)
**Fibrinogen (mg/L)**	3470 (2910-3980)	3230 (2660-3600)	3180 (2690-3720)
**Plasminogen (%)**	100 (88-114)	96 (87-107)	100 (88-109)

Characteristics are expressed as numbers or as medians with interquartile range. A detailed overview of the rare coagulation factor deficiency subgroups is provided in Supplementary Table 3. ISTH-BAT, International Society on Thrombosis and Haemostasis Bleeding Assessment Tool; PAI-1, plasminogen activator inhibitor 1.

### 3.2 *SERPINE1* coding variants

Informed consent for exome sequencing was obtained from 142 patients, including 125 with RCFDs, 10 with PAI-1 deficiency, and 7 with isolated hyperfibrinolysis. Heterozygous *SERPINE1* variants were identified in 30 of 125 RCFD patients (24%), with A15T (rs6092) and V17I (rs6090) being the most frequent, at allele frequencies closely matching those in the general population (Table 2); one patient carried both variants, accounting for the 31 variants identified in total. The V31M and N232S variants were each observed once. In the PAI-1 deficiency and isolated hyperfibrinolysis groups, one variant carrier was identified in each group. No overrepresentation of *SERPINE1* variants was observed in patients with PAI-1 deficiency or isolated hyperfibrinolysis.

**Table 2:** Occurrence of *SERPINE1* coding variants in patients with PAI-1 deficiency, isolated hyperfibrinolysis, and rare coagulation factor deficiencies, compared to allele frequencies in the general population.

*SERPINE1* variant	PAI-1 deficiency (n = 10)	Isolated hyperfibrinolysis (n = 7)	Rare coagulation factor deficiencies (n = 125)	General population
**A15T p.Ala15Thr** **rs6092**	1 (10%) AF 0.050	-	25 (20%) AF 0.100	AF 0.104
**V17I p.Val17Ile** **rs6090**	-	1 (14%) AF 0.071	4 (4%) AF 0.016	AF 0.017
**V31M p.Val31Met** **rs141347752**	-	-	1 (1%) AF 0.004	AF 0.00043
**N232S p.Asn232Ser** **rs147003064**	-	-	1 (1%) AF 0.004	AF 0.00013

Variant frequencies are expressed as number of carriers (percentage of sequenced patients) and allele frequency (AF). General population allele frequencies were obtained from the Genome Aggregation Database (gnomAD) v4.1. AF, allele frequency; PAI-1, plasminogen activator inhibitor 1.

**Figure 1:**
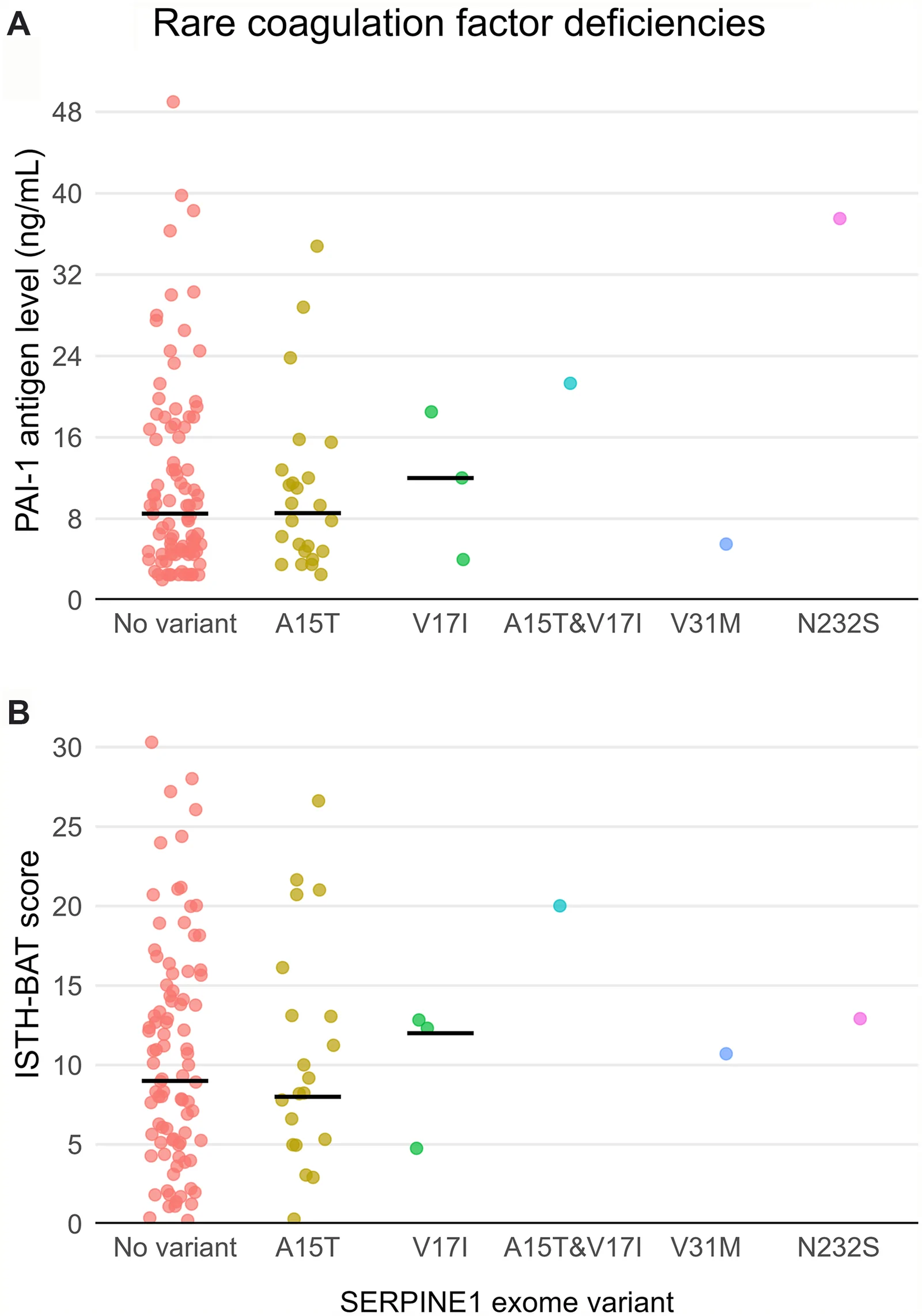
PAI-1 antigen levels (A) and ISTH-BAT scores (B) in RBiN study patients with rare coagulation factor deficiencies (n = 125), stratified by *SERPINE1* exome variants. ISTH-BAT, International Society on Thrombosis and Haemostasis Bleeding Assessment Tool; PAI-1, plasminogen activator inhibitor 1.

**Figure 2:**
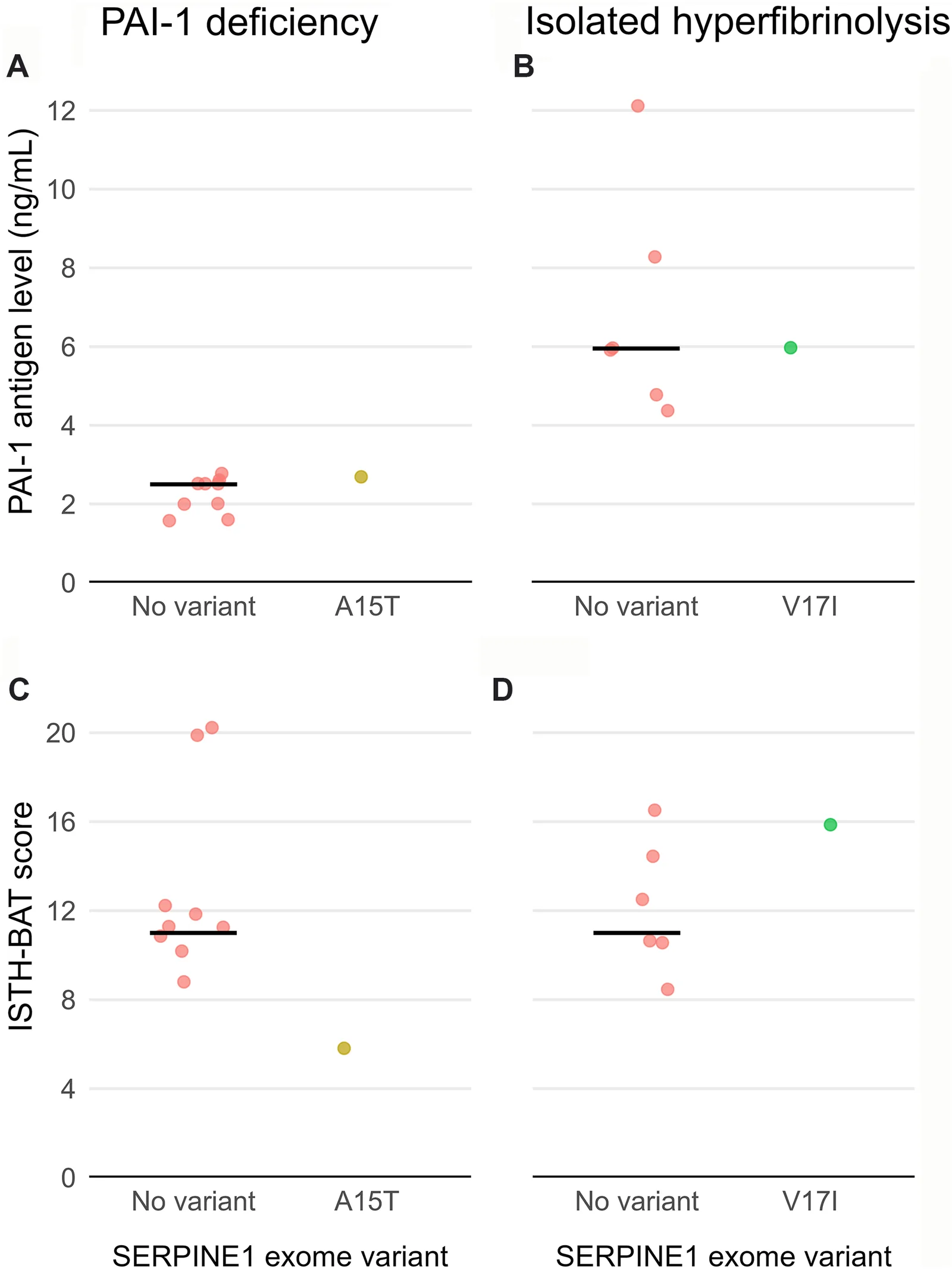
PAI-1 antigen levels and ISTH-BAT scores in RBiN study patients with PAI-1 deficiency (n = 10, panels A & C) or isolated hyperfibrinolysis (n = 7, panels B & D), stratified by *SERPINE1* exome variants. ISTH-BAT, International Society on Thrombosis and Haemostasis Bleeding Assessment Tool; PAI-1, plasminogen activator inhibitor 1.

The presence of an isolated A15T or V17I variant was not associated with lower PAI-1 antigen levels. In the RCFD group, median PAI-1 antigen levels were 8.6 (IQR, 4.8-12.2) ng/mL in A15T carriers and 12.0 (8.0-15.2) ng/mL in V17I carriers, compared with 8.5 (4.7-17.0) ng/mL in RCFD patients without a *SERPINE1* variant (Figure 1A). ISTH-BAT scores were also similar across variant subgroups, with median scores of 8 (5-13) in A15T carriers, 12 (8.5-12.5) in V17I carriers, and 9 (5-15) in patients without a variant (Figure 1B). In patients with PAI-1 deficiency or isolated hyperfibrinolysis, only 2 patients carried a *SERPINE1* variant, precluding meaningful calculation of median PAI-1 antigen or ISTH-BAT scores (Figure 2).

### 3.3 Other variants

For patients with PAI-1 deficiency or isolated hyperfibrinolysis, no pathogenic, likely pathogenic, or variants of uncertain significance were identified in 156 other genes involved in hemostasis. This analysis also included genes related to the fibrinolytic system (eg, *PLG, PLAT, PLAU,* and *SERPINF2*).

### 3.4 *SERPINE1* promoter polymorphisms

The distribution of *SERPINE1* promoter polymorphisms across study groups is shown in Table 3. The 5G allele frequency was 0.50 in patients with PAI-1 deficiency and 0.79 in patients with isolated hyperfibrinolysis, compared to 0.56 in the general population. The -844G allele frequency was 0.45 and 0.57 in patients with PAI-1 deficiency and isolated hyperfibrinolysis, respectively, compared to 0.53 in the general population. These differences were not statistically significant.

**Table 3:** Distribution of *SERPINE1* promoter polymorphisms in patients with PAI-1 deficiency and isolated hyperfibrinolysis.

*SERPINE1* promoter polymorphism	PAI-1 deficiency (n=10)	Isolated hyperfibrinolysis (n=7)	General population
**c.-675 4G/5G** **rs1799889**	4G/4G: 2 (20%) 4G/5G: 6 (60%) 5G/5G: 2 (20%) AF (5G): 0.50	4G/4G: 0 (0%) 4G/5G: 3 (43%) 5G/5G: 4 (57%) AF (5G): 0.79	AF (5G): 0.56
**c.-844 A>G** **rs2227631**	AA: 2 (20%) AG: 7 (70%) GG:1 (10%) AF (G): 0.45	AA: 1 (14%) AG: 4 (57%) GG: 2 (29%) AF (G): 0.57	AF (G): 0.53

Genotype frequencies are expressed as number of patients (percentage of genotyped patients). General population allele frequencies were obtained from the Genome Aggregation Database (gnomAD) v4.1. AF, allele frequency; PAI-1, plasminogen activator inhibitor 1.

**Figure 3:**
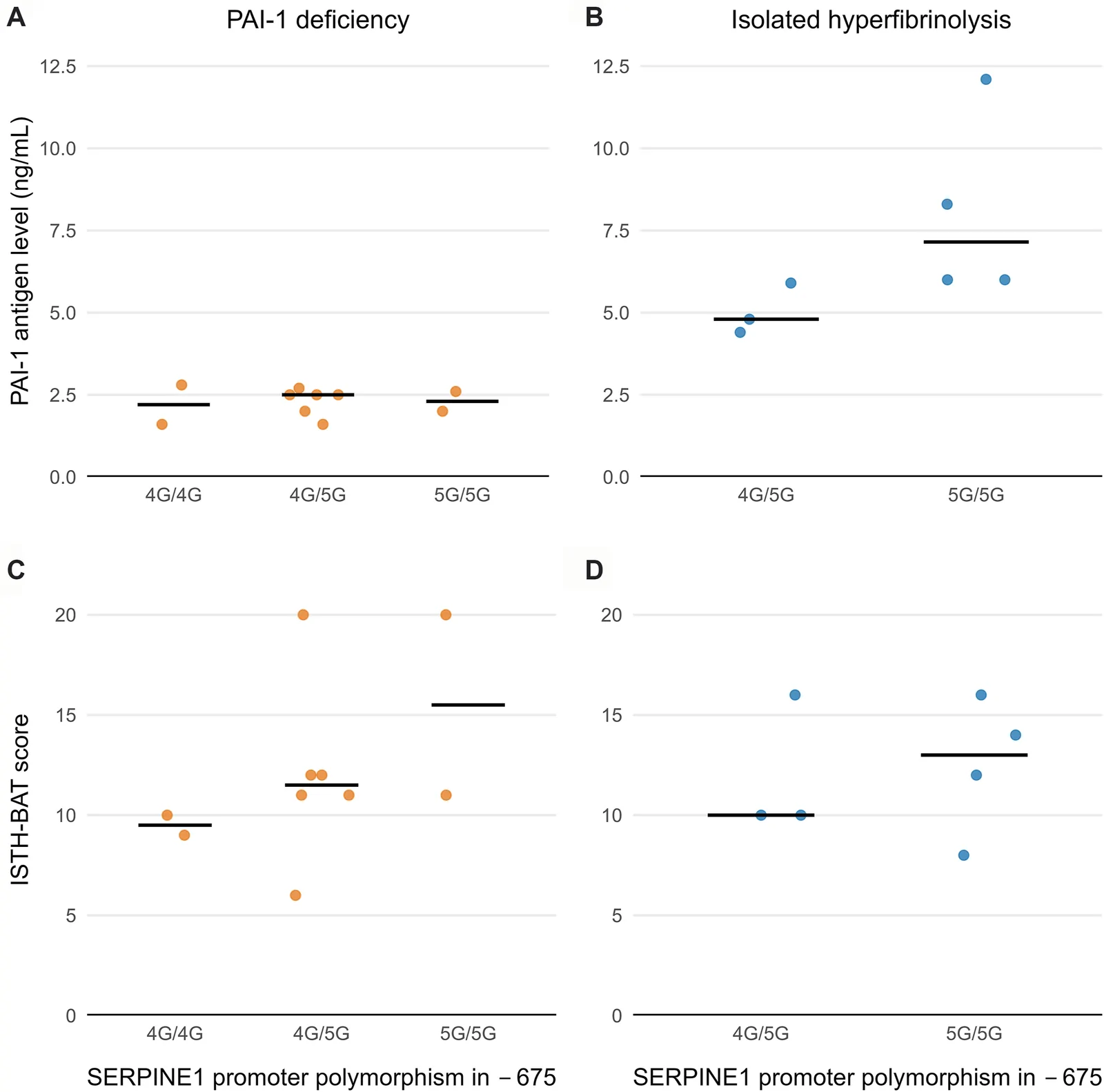
PAI-1 antigen levels and ISTH-BAT scores in RBiN study patients with PAI-1 deficiency (n = 10, panels A & C) or isolated hyperfibrinolysis (n = 7, panels B & D), stratified by *SERPINE1* -675 4G/5G promoter polymorphism. ISTH-BAT, International Society on Thrombosis and Haemostasis Bleeding Assessment Tool; PAI-1, plasminogen activator inhibitor 1.

PAI-1 antigen levels did not differ among the -675 promoter polymorphisms (4G/4G, 4G/5G, and 5G/5G) in patients with PAI-1 deficiency (Figure 3A). In patients with isolated hyperfibrinolysis, those carrying the 5G/5G genotype tended to have higher PAI-1 antigen levels than individuals with the 4G/5G genotype (Figure 3B), although this difference was not statistically significant (*P* = .14). Across both patient groups, median ISTH-BAT scores varied between 9.5 and 15.5 (Figure 3C, D), with no significant differences observed.

**Figure 4:**
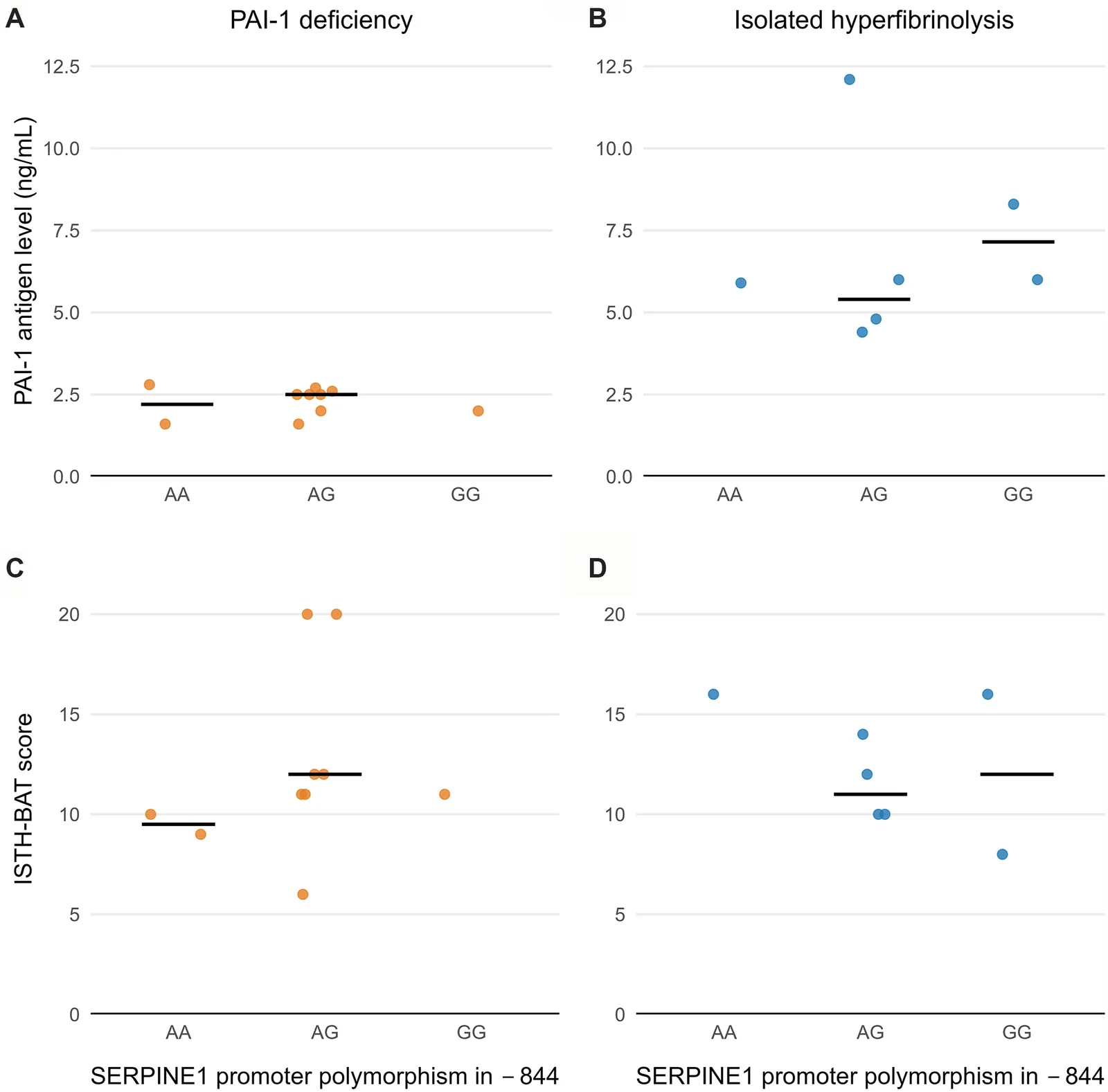
PAI-1 antigen levels and ISTH-BAT scores in RBiN study patients with PAI-1 deficiency (n = 10, panels A & C) or isolated hyperfibrinolysis (n = 7, panels B & D), stratified by *SERPINE1* -844 A>G promoter polymorphism. ISTH-BAT, International Society on Thrombosis and Haemostasis Bleeding Assessment Tool; PAI-1, plasminogen activator inhibitor 1.

PAI-1 antigen levels also did not differ significantly among the -844 promoter polymorphisms (AA, AG, and GG) in patients with PAI-1 deficiency or isolated hyperfibrinolysis (Figure 4A, B). Median ISTH-BAT scores ranged from 9.5 to 12 across the 3 genotypes, with no significant differences observed between polymorphisms or disorders (Figure 4C, D).

### 3.5 Plasmin generation assay

**Figure 5:**
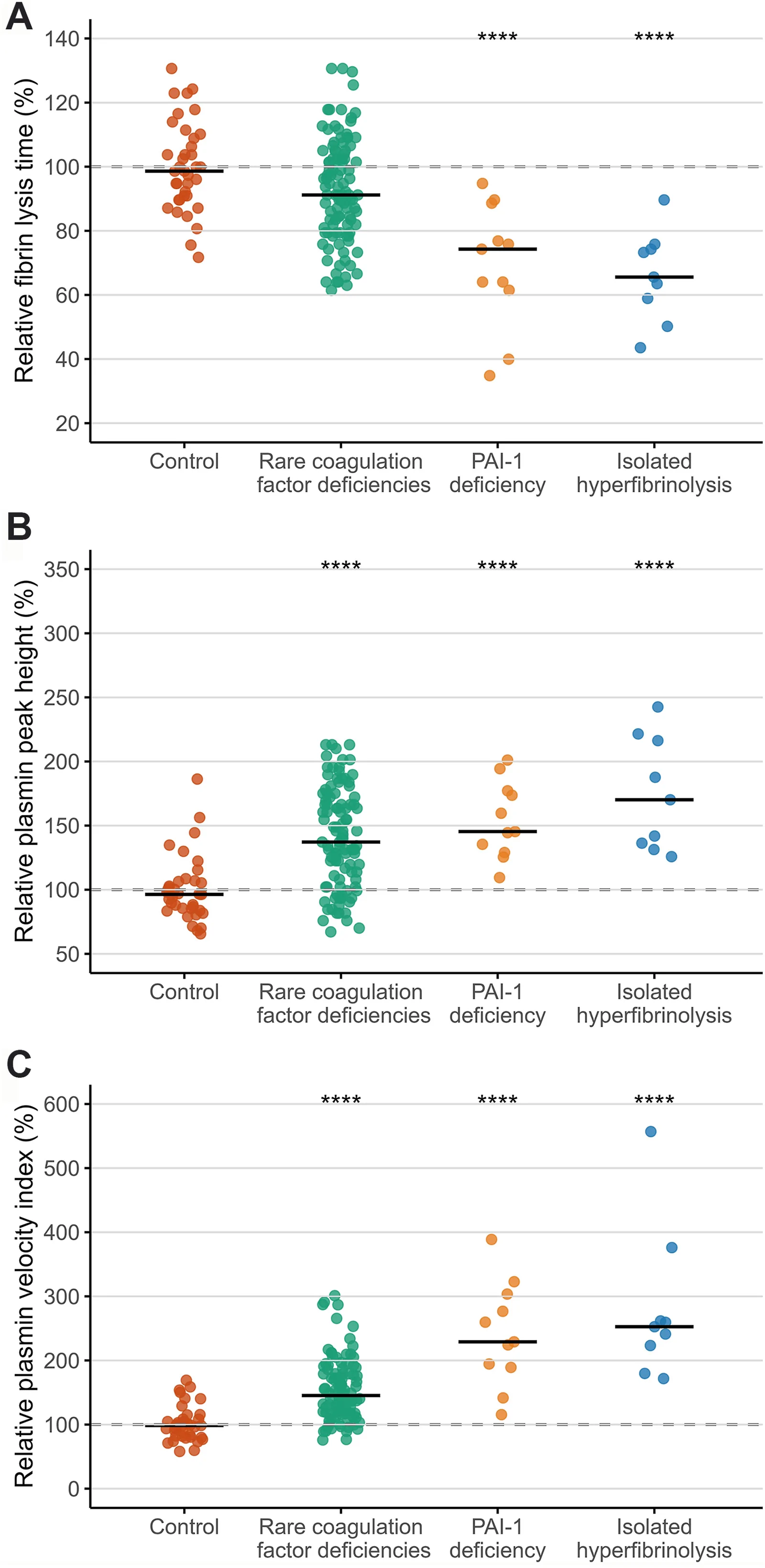
Relative Fibrin lysis times (A), relative plasmin peak heights (B), and relative plasmin velocity indices (C) of patients with rare coagulation factor deficiencies (n = 107), PAI-1 deficiency (n = 11), or isolated hyperfibrinolysis (n = 9), relative to the mean of a cohort of 37 healthy controls. PAI-1, plasminogen activator inhibitor 1.

Patients with PAI-1 deficiency and isolated hyperfibrinolysis both showed significantly enhanced plasmin generation. This was reflected by significantly shorter median fibrin lysis times (74% and 66% of the average value of healthy controls, respectively, Figure 5A) and higher median plasmin peak heights (145% and 170%; Figure 5B). The combination of both parameters resulted in a median plasmin velocity index of 229% and 253%, respectively (Figure 5C). The group with RCFDs showed a subtle, nonsignificant decrease in fibrin lysis time (median 91%). In this group, the median plasmin peak and plasmin velocity index were significantly elevated (137% and 145%), although the increase was limited compared to the groups with hyperfibrinolysis. Fibrin lysis times, plasmin peak heights, and plasmin velocity indices did not correlate with PAI-1 antigen levels, ISTH-BAT scores, or the presence of specific *SERPINE1* variants.

## 4 Discussion

In this study, we investigated coding variants and 2 common promoter polymorphisms (-675 4G/5G and -844A>G) in the *SERPINE1* gene in patients with PAI-1 deficiency, isolated hyperfibrinolysis, and RCFDs. Heterozygous coding variants were rare in patients with PAI-1 deficiency and isolated hyperfibrinolysis, and were limited to the common polymorphisms A15T and V17I. These polymorphisms were also observed in patients with RCFDs. None of these variants were associated with PAI-1 antigen levels or bleeding phenotype. Similarly, the promoter polymorphisms -675 4G/5G and -844A>G showed no consistent association with PAI-1 antigen levels or ISTH-BAT scores. Together, these findings indicate that PAI-1 deficiency and isolated hyperfibrinolysis is not driven by common coding variants or promoter polymorphisms in *SERPINE1*.

Additionally, patients with PAI-1 deficiency or isolated hyperfibrinolysis showed significantly shorter fibrin lysis times, higher plasmin peak heights, and higher plasmin velocity indices than healthy controls. However, these parameters did not correlate with PAI-1 antigen levels, ISTH-BAT scores, or specific *SERPINE1* variants or promoter polymorphisms.

### 4.1 Homozygous coding variants

Our findings are in line with previous reports, as only a small number of pathogenic *SERPINE1* variants have been confirmed to date, and these variants are considered autosomal recessive [6,17]. Notably, we did not identify any homozygous coding variants in our cohort.

The first causal variant was described by Fay et al. [14] in 1992, who reported a homozygous 2-base pair insertion (c.699_700dupTA) in exon 4 of *SERPINE1*, causing a frameshift and premature stop codon (p.Thr234fs). The resulting truncated protein lacked the reactive center and was unstable, leading to a complete absence of detectable PAI-1 activity and antigen. Family analysis revealed 7 homozygous individuals, all with a clear bleeding tendency, and 19 heterozygous carriers without abnormal bleeding, even after major trauma or surgery [6]. In the broader Old Order Amish community surrounding this family, more than 100 individuals have been identified as heterozygous carriers of this variant, none of whom have experienced bleeding symptoms [4]. These findings are consistent with an autosomal recessive mode of inheritance for pathogenic *SERPINE1* variants. In line with these observations, Iwaki et al. [17] reported in 2011 a homozygous single-nucleotide duplication in exon 3 (c.356dupC; p.Ile120Aspfs∗42), resulting in a frameshift and premature stop. This variant was associated with complete functional deficiency and bleeding, while bleeding symptoms were not reported in heterozygous family members. In 2017, the same group described a homozygous missense variant in exon 9 (c.1189G>C; p.Gly397Arg) affecting a highly conserved glycine in strand 5B of the β-sheet. This mutation caused intracellular polymerization of PAI-1, impairing its secretion and resulting in a marked PAI-1 deficiency (0.12 ng/mL) and severe bleeding [18]. Together, these findings indicate that homozygous truncating or missense variants in *SERPINE1* are associated with PAI-1 deficiency and a corresponding bleeding phenotype, consistent with autosomal recessive inheritance.

### 4.2 Heterozygous coding variants

Several studies have linked heterozygous missense variants to PAI-1 deficiency and bleeding phenotypes. Zhang et al. [15] described the A15T substitution, which affects the central hydrophobic core of the PAI-1 signal peptide, in a patient with reduced PAI-1 levels and prolonged bleeding after trauma or surgery. However, the reported patient also had an isolated prolonged prothrombin time with FVII unmeasured, such that a concomitant factor FVII deficiency could not be excluded. Furthermore, an *in vitro* model using Chinese hamster ovary cells in the same study showed no significant difference in PAI-1 antigen levels between wild-type and A15T-carrying cells [15]. A second study associated A15T with increased bleeding in a patient with normal PAI-1 antigen but reduced PAI-1 activity [16], though these measurements were obtained using a nonstandardized thromboelastographic assay not specific for PAI-1 activity, in which values of zero can still fall within the normal reference range [4,12,29]. The same research group also linked V17I, located in the same central hydrophobic core region of the signal peptide, to PAI-1 deficiency based solely on *in silico* analysis without clinical evidence [29]. Based on our data and other studies, it appears unlikely that these 2 heterozygous missense variants are truly causal for PAI-1 deficiency. These variants are common polymorphisms in the general population, with heterozygous carrier frequencies of approximately 19% to 21% and 3%, respectively [30,31]. Moreover, our study shows that these variants are not overrepresented in patients with PAI-1 deficiency compared to individuals with other bleeding disorders, and that they are not associated with lower PAI-1 antigen levels or higher ISTH-BAT scores.

### 4.3 Promoter polymorphisms

Previous studies have also explored the impact of *SERPINE1* promoter polymorphisms on PAI-1 levels and coagulation function. The 4G/4G genotype of the -675 4G/5G promoter polymorphism has since 1995 been associated with higher PAI-1 levels and an increased risk of myocardial infarction, stroke, and venous thromboembolism [[32], [33], [34], [35]]. However, most large studies have concluded that this association is neither independent of other cardiovascular risk factors nor consistent across populations [[36], [37], [38], [39], [40], [41], [42]]. In addition to the -675 4G/5G variant, the -844 A>G polymorphism is frequently observed in the PAI-1 promoter, but it likewise appears not to affect PAI-1 antigen levels or thrombotic risk [30,[43], [44], [45]]. Several reports have nonetheless suggested that both promoter polymorphisms may augment thrombotic risk in carriers of the factor (F)V Leiden or prothrombin G20210A variants [[46], [47], [48], [49]]. Despite its association with higher PAI-1 levels, the -675 4G/4G genotype was also observed in patients with PAI-1 deficiency and a clinically relevant bleeding phenotype in our cohort. Overall, we observed no significant differences in PAI-1 antigen levels or bleeding tendency between the genotypes, a pattern that was similarly seen for the -844 A>G variant.

### 4.4 Plasmin generation assays

Enhanced plasmin generation was observed in patients with PAI-1 deficiency, consistent with the increased fibrinolytic activity expected in this condition. Patients with isolated hyperfibrinolysis, diagnosed based on an elevated euglobin clot lysis time ratio, also exhibited enhanced plasmin generation, at a similar level to patients with PAI-1 deficiency. The increased plasmin generation in these groups appears to be more modest than that observed in patients with α2-antiplasmin deficiency from the same cohort [50]. These data are shown in Supplementary Figure 2 for reference. Our results are consistent with previous research, which found α2-antiplasmin to be a more potent inhibitor of fibrinolysis than PAI-1 [51]. Patients with RCFDs also showed mildly elevated plasmin generation, primarily reflected in an increased plasmin peak height. This may be explained by reduced thrombin generation in this group, leading to decreased activation of thrombin-activatable fibrinolysis inhibitor (TAFI) and consequently lower TAFI activity available to inhibit plasmin generation [[51], [52], [53]]. Van Geffen et al. [27] demonstrated that the novel hemostasis assay is particularly sensitive to TAFI-mediated inhibition of fibrinolysis, such that even modest reductions in thrombin generation are reflected in the plasmin generation parameters [27].

We did not observe any correlation between the extent of enhanced plasmin generation (ie, reduced fibrin lysis time and/or increased plasmin peak height) and PAI-1 antigen levels or bleeding tendency as assessed by ISTH-BAT scores. The absence of a correlation with PAI-1 antigen levels may reflect the complex, multifactorial regulation of fibrinolysis, in which small reductions in PAI-1 at low concentrations may have disproportionate effects on fibrinolytic activity. The absence of a correlation with ISTH-BAT scores may partly reflect limitations of the ISTH-BAT score, which depends on patient reporting (potentially over- or underestimating bleeding) and on the hemostatic challenges each patient has experienced, including trauma, surgery, or childbirth. Technical and biological variation in plasmin generation measurements, as well as the potential influence of other fibrinolytic regulators, may have further contributed. Indeed, substantial interindividual variation in plasmin generation parameters has previously been observed among patients with complete PAI-1 deficiency carrying an identical homozygous *SERPINE1* mutation, indicating that plasmin generation as measured by the novel hemostasis assay does not straightforwardly reflect PAI-1 status [12]. Finally, the limited number of patients included in this study may have reduced our ability to detect correlations.

### 4.5 Bleeding disorders of unknown cause

The overlap between PAI-1 deficiency or isolated hyperfibrinolysis and bleeding disorders of unknown cause (BDUC) warrants explicit consideration. Several studies have identified hyperfibrinolytic features in subsets of patients with BDUC, including evidence of reduced PAI-1 antigen levels as a potential underlying mechanism [[54], [55], [56], [57]]. We propose that the directionality of this relationship is important: rather than classifying patients with low PAI-1 levels or hyperfibrinolysis as BDUC, it is more likely that a subset of patients currently labeled as having BDUC have an underlying fibrinolytic disorder that remains undetected due to the limited availability of validated fibrinolysis assays in routine clinical practice. The present findings may contribute to better characterization of this underdiagnosed patient population.

### 4.6 Clinical implications, strengths, and limitations

This study represents a distinct cohort of patients with confirmed PAI-1 deficiency, all presenting with a clear bleeding tendency and reduced PAI-1 antigen levels as measured according to current clinical standards. By combining detailed genotyping, plasmin generation measurements, and standardized bleeding assessments, we provide a comprehensive evaluation of the presence of variants in the *SERPINE1* gene and the potential influence on clinical and fibrinolytic characteristics. Our findings underscore that measurement of PAI-1 antigen remains the primary method to identify PAI-1 deficiency as *SERPINE1* sequencing rarely reveals pathogenic variants. Genetic testing may, however, provide value in research settings or for confirmatory purposes in families with severe or recurrent deficiency. The main limitation of this work lies in the low absolute number of patients with PAI-1 deficiency and isolated hyperfibrinolysis, which is inevitable given the rarity of the disorder. An additional limitation is that a small subset of the comparator patient group with RCFDs did not have documented bleeding symptoms, and it cannot be excluded that mildly reduced factor levels in these cases reflect normal biological variation rather than a clinically relevant deficiency. Nonetheless, the majority of patients in this comparator group had a clear bleeding tendency, supporting the clinical relevance of the diagnoses in this group overall. Moreover, whole-exome sequencing was performed with high quality and coverage, focusing on a curated panel of genes involved in hemostasis and fibrinolysis. Additional analyses for copy-number variation and structural rearrangements did not reveal missed coding changes, making it unlikely that coding variants were missed. The absence of pathogenic coding variants in this well-characterized cohort might reflect strong negative selection against complete loss-of-function mutations in *SERPINE1*, or the involvement of noncoding, regulatory, or epigenetic mechanisms that remain to be elucidated.

### 4.7 Future directions

Building on these observations, further research should investigate mechanisms beyond coding variants that may underlie PAI-1 deficiency. Variants in untranslated or intronic regions, as well as in enhancers or microRNA-binding sites within the 3’ UTR (eg, *miR-617, miR-30c,* and *miR-421*), may influence *SERPINE1* expression through transcriptional, splicing-related, or post-transcriptional mechanisms without altering the coding sequence [58,59]. Epigenetic regulation through promoter methylation has also been shown in mice to modulate *Serpine1* transcription under certain conditions [60]. Elucidating such mechanisms may clarify why some individuals show markedly reduced PAI-1 antigen levels despite the absence of coding variants.

Beyond *SERPINE1*, additional molecular pathways may influence PAI-1 expression, stability, or activity. Variants in proteins outside current hemostasis panels, as well as variants that disrupt interactions with *SERPINE1*, could contribute to reduced PAI-1 antigen levels even when no coding variants in *SERPINE1* are present. Future research should therefore adopt a systems-level approach. Whole-genome sequencing and transcriptomic profiling in well-phenotyped cohorts may uncover noncoding or regulatory mechanisms driving PAI-1 deficiency. Functional studies, including luciferase reporter studies of patient-specific promoter variants and cellular models exploring PAI-1 secretion and stability, could clarify the roles of transcriptional and posttranslational regulation. Finally, integrating multiomics data, including transcriptomics, epigenomics, and proteomics, with plasmin generation measurements across international registries will be essential to identify novel regulatory factors. This approach will also provide functional insight into fibrinolysis, complementing the understanding of the genetic and molecular determinants of PAI-1 deficiency.

### 4.8 Conclusions

Genetic analysis of clinically significant PAI-1 deficiency and isolated hyperfibrinolysis in this study identified common coding variants and promoter polymorphisms in *SERPINE1*. However, none were associated with PAI-1 antigen levels or bleeding tendency, and their frequencies did not exceed those observed in patients with other rare bleeding disorders and the general population. Measurement of PAI-1 antigen levels under strict conditions remains the cornerstone for diagnosis, while genetic testing is mainly useful in research settings or for confirming severe familial cases. Plasmin generation assays, such as the Nijmegen Hemostasis Assay, effectively detect enhanced plasmin generation in patients with PAI-1 deficiency or isolated hyperfibrinolysis. Future studies should explore noncoding, regulatory, and epigenetic mechanisms, as well as broader molecular pathways, to fully elucidate the determinants of PAI-1 deficiency and the associated effects on plasmin generation and bleeding phenotype.

## References

[1] Saes J.L., Schols S.E.M., van Heerde W.L., Nijziel M.R. (2018). Hemorrhagic disorders of fibrinolysis: a clinical review. J Thromb Haemost.

[2] Sillen M., Declerck P.J. (2021). A narrative review on plasminogen activator inhibitor-1 and its (patho)physiological role: to target or not to target?. Int J Mol Sci.

[3] Heiman M., Gupta S., Shapiro A.D. (2014). The obstetric, gynaecological and fertility implications of homozygous PAI-1 deficiency: single-centre experience. Haemophilia.

[4] Mehta R., Shapiro A.D. (2008). Plasminogen activator inhibitor type 1 deficiency. Haemophilia.

[5] Minowa H., Takahashi Y., Tanaka T., Naganuma K., Ida S., Maki I. (1999). Four cases of bleeding diathesis in children due to congenital plasminogen activator inhibitor-1 deficiency. Haemostasis.

[6] Fay W.P., Parker A.C., Condrey L.R., Shapiro A.D. (1997). Human plasminogen activator inhibitor-1 (PAI-1) deficiency: characterization of a large kindred with a null mutation in the PAI-1 gene. Blood.

[7] Morimoto Y., Yoshioka A., Imai Y., Takahashi Y., Minowa H., Kirita T. (2004). Haemostatic management of intraoral bleeding in patients with congenital deficiency of alpha2-plasmin inhibitor or plasminogen activator inhibitor-1. Haemophilia.

[8] Rughani A.I., Holmes C.E., Penar P.L. (2009). A novel association between a chronic subdural hematoma and a fibrinolytic pathway defect: case report. Neurosurgery.

[9] Matsui H., Takahashi Y., Matsunaga T., Tanaka-Horie T., Minowa H., Sugimoto M. (2001). Successful arthroscopic treatment of pigmented villonodular synovitis of the knee in a patient with congenital deficiency of plasminogen activator inhibitor-1 and recurrent haemarthrosis. Haemostasis.

[10] Prabhudesai A., Sharma R., Shetty S., Phadnis A., Kulkarni B. (2020). Congenital PAI-1 deficiency results in psoas hematoma in an Indian patient. Thromb Res.

[11] Angleton P., Chandler W.L., Schmer G. (1989). Diurnal variation of tissue-type plasminogen activator and its rapid inhibitor (PAI-1). Circulation.

[12] Saes J.L., Schols S.E.M., Betbadal K.F., van Geffen M., Verbeek-Knobbe K., Gupta S. (2019). Thrombin and plasmin generation in patients with plasminogen or plasminogen activator inhibitor type 1 deficiency. Haemophilia.

[13] Heiman M., Gupta S., Khan S., Vaughan D., Shapiro A., Adam M.P., Bick S., Mirzaa G.M. (2017). GeneReviews®.

[14] Fay W.P., Shapiro A.D., Shih J.L., Schleef R.R., Ginsburg D. (1992). Brief report: complete deficiency of plasminogen-activator inhibitor type 1 due to a frame-shift mutation. N Engl J Med.

[15] Zhang Z.Y., Wang Z.Y., Dong N.Z., Bai X., Zhang W., Ruan C.G. (2005). A case of deficiency of plasma plasminogen activator inhibitor-1 related to Ala15Thr mutation in its signal peptide. Blood Coagul Fibrinolysis.

[16] Jankun J., Skrzypczak-Jankun E. (2010). Bleeding diathesis is associated with an A15T heterozygous mutation in exon 2 of the plasminogen activator inhibitor type 1. Exp Ther Med.

[17] Iwaki T., Tanaka A., Miyawaki Y., Suzuki A., Kobayashi T., Takamatsu J. (2011). Life-threatening hemorrhage and prolonged wound healing are remarkable phenotypes manifested by complete plasminogen activator inhibitor-1 deficiency in humans. J Thromb Haemost.

[18] Iwaki T., Nagahashi K., Takano K., Suzuki-Inoue K., Kanayama N., Umemura K. (2017). Mutation in a highly conserved glycine residue in strand 5B of plasminogen activator inhibitor 1 causes polymerisation. Thromb Haemost.

[19] Saes J.L., Verhagen M.J.A., Meijer K., Cnossen M.H., Schutgens R.E.G., Peters M. (2020). Bleeding severity in patients with rare bleeding disorders: real-life data from the RBiN study. Blood Adv.

[20] Li H., Durbin R. (2010). Fast and accurate long-read alignment with Burrows-Wheeler transform. Bioinformatics.

[21] McKenna A., Hanna M., Banks E., Sivachenko A., Cibulskis K., Kernytsky A. (2010). The Genome Analysis Toolkit: a MapReduce framework for analyzing next-generation DNA sequencing data. Genome Res.

[22] Willems S.P.E., Simons A., Saes J.L., Weiss M., Rijpma S., Schoormans S. (2024). Targeted exome analysis in patients with rare bleeding disorders: data from the Rare Bleeding disorders in the Netherlands study. Res Pract Thromb Haemost.

[23] Downes K., Megy K., Duarte D., Vries M., Gebhart J., Hofer S. (2019). Diagnostic high-throughput sequencing of 2396 patients with bleeding, thrombotic, and platelet disorders. Blood.

[24] Megy K., Downes K., Simeoni I., Bury L., Morales J., Mapeta R. (2019). Curated disease-causing genes for bleeding, thrombotic, and platelet disorders: communication from the SSC of the ISTH. J Thromb Haemost.

[25] Krumm N., Sudmant P.H., Ko A., O'Roak B.J., Malig M., Coe B.P. (2012). Copy number variation detection and genotyping from exome sequence data. Genome Res.

[26] Richards S., Aziz N., Bale S., Bick D., Das S., Gastier-Foster J. (2015). Standards and guidelines for the interpretation of sequence variants: a joint consensus recommendation of the American College of Medical Genetics and Genomics and the Association for Molecular Pathology. Genet Med.

[27] van Geffen M., Loof A., Lap P., Boezeman J., Laros-van Gorkom B.A., Brons P. (2011). A novel hemostasis assay for the simultaneous measurement of coagulation and fibrinolysis. Hematology.

[28] Haisma B., Schols S.E.M., van Oerle R.G.M., Verbeek-Knobbe K., Hellenbrand D., Verwoerd E.J. (2024). Comparative analysis of thrombin generation platforms for patients with coagulation factor deficiencies: a comprehensive assessment. Thromb Res.

[29] Jankun J., Skrzypczak-Jankun E. (2011). Val17Ile single nucleotide polymorphisms similarly as Ala15Thr could be related to the lower secretory dynamics of PAI-1 secretion: theoretical evidence. Curr Mol Med.

[30] Lopes C., Dina C., Durand E., Froguel P. (2003). PAI-1 polymorphisms modulate phenotypes associated with the metabolic syndrome in obese and diabetic Caucasian population. Diabetologia.

[31] GnomAD browser SERPINE1 serpin family E member 1.. https://gnomad.broadinstitute.org/gene/ENSG00000106366?dataset=gnomad_r4.

[32] Panahloo A., Mohamed-Ali V., Lane A., Green F., Humphries S.E., Yudkin J.S. (1995). Determinants of plasminogen activator inhibitor 1 activity in treated NIDDM and its relation to a polymorphism in the plasminogen activator inhibitor 1 gene. Diabetes.

[33] Eriksson P., Kallin B., van‘t Hooft F.M., Båvenholm P., Hamsten A. (1995). Allele-specific increase in basal transcription of the plasminogen-activator inhibitor 1 gene is associated with myocardial infarction. Proc Natl Acad Sci U S A.

[34] Bang C.O., Park H.K., Ahn M.Y., Shin H.K., Hwang K.Y., Hong S.Y. (2001). 4G/5G polymorphism of the plasminogen activator inhibitor-1 gene and insertion/deletion polymorphism of the tissue-type plasminogen activator gene in atherothrombotic stroke. Cerebrovasc Dis.

[35] Sartori M.T., Wiman B., Vettore S., Dazzi F., Girolami A., Patrassi G.M. (1998). 4G/5G polymorphism of PAI-1 gene promoter and fibrinolytic capacity in patients with deep vein thrombosis. Thromb Haemost.

[36] van Goor M.L., Gómez García E., Leebeek F., Brouwers G.J., Koudstaal P., Dippel D. (2005). The plasminogen activator inhibitor (PAI-1) 4G/5G promoter polymorphism and PAI-1 levels in ischemic stroke. A case-control study. Thromb Haemost.

[37] Anderson J.L., Muhlestein J.B., Habashi J., Carlquist J.F., Bair T.L., Elmer S.P. (1999). Lack of association of a common polymorphism of the plasminogen activator inhibitor-1 gene with coronary artery disease and myocardial infarction. J Am Coll Cardiol.

[38] Doggen C.J., Bertina R.M., Cats V.M., Reitsma P.H., Rosendaal F.R. (1999). The 4G/5G polymorphism in the plasminogen activator inhibitor-1 gene is not associated with myocardial infarction. Thromb Haemost.

[39] Catto A.J., Carter A.M., Stickland M., Bamford J.M., Davies J.A., Grant P.J. (1997). Plasminogen activator inhibitor-1 (PAI-1) 4G/5G promoter polymorphism and levels in subjects with cerebrovascular disease. Thromb Haemost.

[40] Ridker P.M., Hennekens C.H., Lindpaintner K., Stampfer M.J., Miletich J.P. (1997). Arterial and venous thrombosis is not associated with the 4G/5G polymorphism in the promoter of the plasminogen activator inhibitor gene in a large cohort of US men. Circulation.

[41] Stegnar M., Uhrin P., Peternel P., Mavri A., Salobir-Pajnic B., Stare J. (1998). The 4G/5G sequence polymorphism in the promoter of plasminogen activator inhibitor-1 (PAI-1) gene: relationship to plasma PAI-1 level in venous thromboembolism. Thromb Haemost.

[42] Agren A., Wiman B., Stiller V., Lindmarker P., Sten-Linder M., Carlsson A. (2006). Evaluation of low PAI-1 activity as a risk factor for hemorrhagic diathesis. J Thromb Haemost.

[43] Grubic N., Stegnar M., Peternel P., Kaider A., Binder B.R. (1996). A novel G/A and the 4G/5G polymorphism within the promoter of the plasminogen activator inhibitor-1 gene in patients with deep vein thrombosis. Thromb Res.

[44] Henry M., Chomiki N., Scarabin P.Y., Alessi M.C., Peiretti F., Arveiler D. (1997). Five frequent polymorphisms of the PAI-1 gene: lack of association between genotypes, PAI activity, and triglyceride levels in a healthy population. Arterioscler Thromb Vasc Biol.

[45] Saidi S., Slamia L.B., Mahjoub T., Ammou S.B., Almawi W.Y. (2007). Association of PAI-1 4G/5G and -844G/A gene polymorphism and changes in PAI-1/tPA levels in stroke: a case-control study. J Stroke Cerebrovasc Dis.

[46] Seguí R., Estellés A., Mira Y., España F., Villa P., Falcó C. (2000). PAI-1 promoter 4G/5G genotype as an additional risk factor for venous thrombosis in subjects with genetic thrombophilic defects. Br J Haematol.

[47] Visanji J.M., Seargent J., Tahri D., Croft S.A., Makris M., Preston F.E. (2000). Influence of the -675 4G/5G dimorphism of the plasminogen activator inhibitor 1 promoter on thrombotic risk in patients with factor V Leiden. Br J Haematol.

[48] Yılmaz E., Akar E., Akar N. (2004). Effect of plasminogen activator inhibitor-1 4G/5G polymorphism in Turkish deep vein thromboembolic patients with and without prothrombin 20210 G-A. Turk J Haematol.

[49] Morange P.E., Henry M., Tregouët D., Granel B., Aillaud M.F., Alessi M.C. (2000). The A -844G polymorphism in the PAI-1 gene is associated with a higher risk of venous thrombosis in factor V Leiden carriers. Arterioscler Thromb Vasc Biol.

[50] Haisma B., Rijpma S.R., Cnossen M.H., den Exter P.L., Kruis I.C., Meijer K. (2024). Enhanced thrombin and plasmin generation profiles in alpha-2-antiplasmin-deficient patients: data from the Rare Bleeding disorders in the Netherlands study. Res Pract Thromb Haemost.

[51] Mutch N.J., Thomas L., Moore N.R., Lisiak K.M., Booth N.A. (2007). TAFIa, PAI-1 and alpha-antiplasmin: complementary roles in regulating lysis of thrombi and plasma clots. J Thromb Haemost.

[52] Bajzar L., Morser J., Nesheim M. (1996). TAFI, or plasma procarboxypeptidase B, couples the coagulation and fibrinolytic cascades through the thrombin-thrombomodulin complex. J Biol Chem.

[53] Mutch N.J., Moore N.R., Wang E., Booth N.A. (2003). Thrombus lysis by uPA, scuPA and tPA is regulated by plasma TAFI. J Thromb Haemost.

[54] Ozolina A., Strike E., Jaunalksne I., Krumina A., Bjertnaes L.J., Vanags I. (2012). PAI-1 and t-PA/PAI-1 complex potential markers of fibrinolytic bleeding after cardiac surgery employing cardiopulmonary bypass. BMC Anesthesiol.

[55] Szczepaniak P., Zabczyk M., Undas A. (2015). Increased plasma clot permeability and susceptibility to lysis are associated with heavy menstrual bleeding of unknown cause: a case-control study. PLoS One.

[56] Vries M.J.A., Macrae F., Nelemans P.J., Kuiper G., Wetzels R.J.H., Bowman P. (2019). Assessment and determinants of whole blood and plasma fibrinolysis in patients with mild bleeding symptoms. Thromb Res.

[57] Gebhart J., Kepa S., Hofer S., Koder S., Kaider A., Wolberg A.S. (2017). Fibrinolysis in patients with a mild-to-moderate bleeding tendency of unknown cause. Ann Hematol.

[58] Zhao C., Liu Z. (2021). MicroRNA 617 targeting SERPINE1 inhibited the progression of oral squamous cell carcinoma. Mol Cell Biol.

[59] Marchand A., Proust C., Morange P.E., Lompré A.M., Trégouët D.A. (2012). miR-421 and miR-30c inhibit SERPINE 1 gene expression in human endothelial cells. PLoS One.

[60] Yao D., Zhao J., Zhang Q., Wang T., Ni M., Qi S. (2022). Aberrant methylation of Serpine1 mediates lung injury in neonatal mice prenatally exposed to intrauterine inflammation. Cell Biosci.

