# Supplementary material for: Genetic and hemostatic characterization of PAI-1 deficiency and isolated hyperfibrinolysis: data from the RBiN study
Source: Res Pract Thromb Haemost. 2026 Aug 27;10(6):106928. doi: 10.1016/j.rpth.2026.106928 (PMC13597289; doi:10.1016/j.rpth.2026.106928)
Supplement: Supplementary file 1 [file mmc1.docx]

**Supplementary tables and figures**

**Supplementary Figure S1:** Impression of the first
derivative of the plasmin generation curve and the
parameters fibrin lysis time, plasmin peak height
and plasmin velocity index.

**Supplementary Figure S2: Fibrin lysis times, plasmin peak heights and plasmin
velocity indices of patients with rare coagulation factor deficiencies (n = 107),
PAI-1 deficiency (n = 11), isolated hyperfibrinolysis (n = 9) or α2-antiplasmin
deficiency (n = 18), relative to the mean of a cohort of 37 healthy controls.**
Data for the α2-antiplasmin deficiency (A2AP) group are from a previous study
using the same cohort (Haisma et al. RPTH, 2024 (30)). For visualization purposes,
plasmin peak heights and velocity indices in this group were capped at 340 (n=1)
and 580 (n=3), respectively. PAI-1 = Plasminogen Activator Inhibitor 1.

| **Gene name** | **OMIM phenotype ID** |
| --- | --- |
| A2M | 614036 |
| ABCG5 | 210250 |
| ABCG8 | 210250 |
| ACBD5 | none |
| ACTB | 243310 |
| ACTN1 | 615193 |
| ACVRL1 | 600376 |
| ADAMTS13 | 274150 |
| ANKRD26 | 188000 |
| ANO6 | 262890 |
| AP3B1 | 608233 |
| AP3D1 | 617050 |
| ARPC1B | 617718 |
| BLOC1S3 | 614077 |
| BLOC1S6 | 614171 |
| BRAF | 613706 |
| C3 | 612925 |
| CALR | 187950 |
| CBL | 613563 |
| CD36 | 608404 |
| CD46 | 612922 |
| CDC42 | 616737 |
| CFB | 612924 |
| CFH | 235400 |
| CFHR1 | 235400 |
| CFHR3 | 235400 |
| CFI | 610984, 612923, 615439 |
| CHST14 | 601776 |
| COL1A1 | 130000 |
| COL3A1 | 130050 |
| COL5A1 | 130000 |
| COL5A2 | 130000 |
| CTLA4 | 152700 |
| CYCS | 612004 |
| DGKE | 615008 |
| DIAPH1 | 124900 |
| DNASE1 | 152700 |
| DTNBP1 | 614076 |
| ENG | 187300 |
| EPHB2 | 618462 |
| ETV6 | 616216 |
| F10 | 227600 |
| F11 | 612416 |
| F12 | 234000 |
| F13A1 | 188050;613225 |
| F13B | 613235 |
| F2 | 188050;613679 |
| F2RL3 | none |
| F5 | 188055;227400 |
| F7 | 227500 |
| F8 | 306700 |
| F9 | 300807;306900 |
| FBN1 | none with bleeding/coagulation |
| FCGR2A | 152700 |
| FCGR2B | 152700 |
| FCGR2C | none |
| FERMT3 | 612840 |
| FGA | 616004;202400 |
| FGB | 616004;202400 |
| FGG | 616004;202400 |
| FLI1 | 147791;188025 |
| FLNA | 300049;300048 |
| FYB1 (=FYB) | 273900 |
| GATA1 | 300835;300367;314050 |
| GATA2 | none with bleeding/coagulation |
| GBA | 231000;231005;230900;230800 |
| GDF2 | 615506 |
| GFI1B | 187900 |
| GGCX | 277450;610842 |
| GNE | none |
| GP1BA | 153670;177820;231200 |
| GP1BB | 231200 |
| GP6 | 614201 |
| GP9 | 231200 |
| HABP2 | 188050 |
| HOXA11 | 605432 |
| HPS1 | 203300 |
| HPS3 | 614072 |
| HPS4 | 614073 |
| HPS5 | 614074 |
| HPS6 | 614075 |
| HRG | 613116 |
| IKZF5 | 619130 |
| ITGA2 | 614200.6213 |
| ITGA2B | 187800;273800;621266 |
| ITGB3 | 619271,621264,619267 |
| JAK2 | 614521 |
| KDSR | 617526 |
| KLKB1 | 612423 |
| KNG1 | 228960 |
| KRAS | 609942 |
| LMAN1 | 227300 |
| LYST | 214500 |
| LZTR1 | 616564 |
| MASTL | 188000 |
| MCFD2 | 613625 |
| MECOM | 616738 |
| MLPH | 609227 |
| MPIG6B (=G6B) | 617441 |
| MPL | 601977;604498 |
| MTHFR | 188050 |
| MYH9 | 155100;603622 |
| MYO5A | 214450 |
| NBEA | none |
| NBEAL2 | 139090 |
| NRAS | 613224 |
| ORAI1 | none |
| P2RY12 | 609821 |
| PIGA | 300818 |
| PLA2G4A | 618372 |
| PLA2G7 | 614278 |
| PLAT | 612348 |
| PLAU | 601709 |
| PLG | 217090 |
| PRKACG | 616176 |
| PROC | 176860;612304 |
| PROS1 | 612336;614514 |
| PROZ | 614024 |
| PTGS1 (COX1) | 605735 |
| PTPN11 | 163950 |
| PTPN22 | 152700 |
| PTPRJ | none |
| RAB27A | 607624 |
| RAF1 | 611553 |
| RASGRP2 | 615888 |
| RBM8A | 274000 |
| RIT1 | 615355 |
| RNU4ATAC | none with bleeding/coagulation |
| RUNX1 | 601399 |
| SERPINC1 | 613118 |
| SERPIND1 (=HCF2) | 612356 |
| SERPINE1 (=PAI1) | 613329 |
| SERPINF2 | 262850 |
| SH2B3 | 187950 |
| SLFN14 | 616913 |
| SOS1 | 610733 |
| SOS2 | 616559 |
| SRC | 616937 |
| STIM1 | 185070 |
| STXBP2 | 613101 |
| TALDO1 | 606003 |
| TBX1 | 192430 |
| TBXA2R | 614009 |
| TBXAS1 | 614158;231095 |
| THBD | 614486 |
| THPO | 187950;620481;620478 |
| TNXB | 606408 |
| TPM4 | 620486 |
| TREX1 | 152700; 225750;610448 |
| TUBB1 | 613112 |
| VIPAS39 | 613404 |
| VKORC1 | 607473 |
| VPS33B | 208085 |
| VWF | 193400;277480;613554 |
| WAS | 301000;313900 |
| WIPF1 | 614493 |

**Supplementary Table S1: Targeted exome sequencing panel
with all analyzed genes involved in thrombosis and hemostasis**

| **Primer** | **Sequence** | **Binding location** |
| --- | --- | --- |
| PCR primer forward | 5’-CCA CTA GTC TAG CCC TAC ATG C-3’ | -1596 to -1574 |
| PCR primer reverse | 5’-TGA GCA AAC CCC AAT AGC CTT-3’ | -217 to -238 |
| Sequencing primer forward | 5’-AAA CGT TGA GGA CCA CTG CTC CAC-3’ | -1036 to -1012 |
| Sequencing primer reverse | 5’-CCA ACA GAG GAC TCT TGG TCT TTC-3’ | -601 to -625 |

**Supplementary Table S2: Primers used for the detection of the -844 A/G and -675 4G/5G
polymorphisms.** Positions of binding locations are relative to the transcription start site of *SERPINE1*
 transcript NM_000602.5. PCR = Polymerase Chain Reaction.

| **Kit** | **Cat#** | **Intra-run CV** | |  | **Inter-run CV** | | | **Lower limit of quantification** |
| --- | --- | --- | --- | --- | --- | --- | --- | --- |
|  |  | High concentration | Low concentration |  | High concentration | Low concentration | |  |
| ZYMUTEST PAI-1 antigen | RK012A | 5.1% | 3.8% |  | 12.3% | | 10.5% | <2.5 ng/mL |
| ZYMUTEST PAI-1 activity | RK019A | 1.3% | 3.4% |  | 4.4% | | 4.1% | <0.5 ng/mL |

**Supplementary Table S3: Performance characteristics of ZYMUTEST PAI-1 antigen and activity assays (Hyphen BioMed, Neuville-sur-Oise, France) as determined by in-house validation.** CV = coefficient of variation.

| Characteristics | Total | Fibrinogen disorders | FII deficiency | FV deficiency | FV/FVIII deficiency | FVII deficiency | FX deficiency | FXI deficiency | FXIII deficiency |
| --- | --- | --- | --- | --- | --- | --- | --- | --- | --- |
| Number of patients | 214 | 42 | 16 | 27 | 6 | 58 | 8 | 43 | 14 |
| Of which female | 124 (58%) | 30 (71%) | 8 (50%) | 16 (59%) | 5 (83%) | 30 (52%) | 6 (75%) | 25 (58%) | 4 (29%) |
| Age (y) | 36 (18–56) | 38 (22-54) | 37 (22-63) | 36 (21-55) | 11 (2-28) | 35 (16-51) | 29 (16-51) | 42 (21-64) | 32 (17-53) |
| Body mass index (kg/m^2^) | 23.7 (20.4–27.3) | 24.8 (21.1-17.1) | 22.8 (20.8-26.4) | 23.9 (21.8-26.0) | 17.4 (17.4-21.8) | 25.5 (21.8-28.7) | 20.9 (19.3-25.1) | 23.9 (18.9-27.2) | 22.9 (20.7-23.9) |
| ISTH-BAT score | 8 (4–14) | 9 (5-13) | 5 (3-13) | 14 (4-19) | 7 (7-13) | 7 (3-11) | 5 (3-8) | 8 (4-11) | 15 (11-22) |
| PAI-1 antigen (ng/mL) | 8.0 (4.5–14.8) | 7.3 (4.8-12.8) | 7.9 (4.3-10.2) | 7.0 (5.3-15.8) | 8.7 (5.6-10.2) | 8.8 (4.2-14.2) | 13.3 (9.5-15.8) | 9.5 (4.5-15.8) | 11.3 (7.0-21.3) |
| Alpha-2-antiplasmin (%) | 109 (100–118) | 117 (108-123) | 103 (99-110) | 112 (103-118) | 117 (110-122) | 105 (97-113) | 111 (105-111) | 108 (102-117) | 109 (102-110) |
| Fibrinogen (mg/L) | 3180 (2690–3720) | 890 (690-1310) | 3250 (2930-3860) | 3280 (2790-3600) | 3280 (3030-3660) | 3230 (2850-3750) | 2830 (2360-3250) | 3310 (2930-4320) | 3060 (2790-3310) |
| Plasminogen (%) | 100 (88–109) | 87 (74-101) | 97 (87-106) | 102 (94-113) | 107 (99-113) | 97 (90-105) | 102 (82-116) | 102 (97-110) | 95 (84-106) |

**Supplementary Table S4: Characteristics of studied patients with rare coagulation factor deficiencies, stratified by deficiency.** Characteristics are expressed as numbers or as medians with interquartile range. ISTH-BAT = International Society on Thrombosis and Hemostasis Bleeding Assessment Tool; PAI-1 = Plasminogen Activator Inhibitor 1.

| Patient | Type | PAI-1 antigen | Gender | ISTH-BAT | Main bleeding presentations† |
| --- | --- | --- | --- | --- | --- |
| 1 | PAI-1 deficiency | 1.5 | Female | 6 | Menorrhagia |
| 2 | PAI-1 deficiency | 1.6 | Female | 20 | Epistaxis, bleeding after tooth extraction and surgery, menorrhagia |
| 3 | PAI-1 deficiency | 1.6 | Female | 10 | Menorrhagia |
| 4 | PAI-1 deficiency | 1.8 | Female | 20 | Menorrhagia, epistaxis, bleeding after surgery and tooth extraction |
| 5 | PAI-1 deficiency | 2.0 | Female | 11 | Bleeding after tooth extraction |
| 6 | PAI-1 deficiency | 2.0 | Female | 11 | Bleeding after tooth extraction and surgery |
| 7 | PAI-1 deficiency | 2.5 | Female | 12 | Bleeding after surgery, postpartum bleeding |
| 8 | PAI-1 deficiency | 2.5 | Female | 11 | Bleeding after surgery |
| 9 | PAI-1 deficiency | 2.5 | Female | 12 | Bleeding after surgery |
| 10 | PAI-1 deficiency | 2.6 | Female | 20 | Menorrhagia, intracerebral bleeding |
| 11 | PAI-1 deficiency | 2.7 | Female | 6 | Bleeding after tooth extraction |
| 12 | PAI-1 deficiency | 2.8 | Female | 9 | Bleeding after surgery |
| 13 | PAI-1 deficiency | 3.1 | Female | 18 | Menorrhagia, postpartum bleeding |
| 14 | PAI-1 deficiency | 3.2 | Female | 20 | Menorrhagia, epistaxis, bleeding after tooth extraction |
| 15 | Isolated hyperfibrinolysis | 3.4 | Female | 11 | Epistaxis, menorrhagia |
| 16 | Isolated hyperfibrinolysis | 3.5 | Female | 10 | Bleeding after surgery, postpartum bleeding |
| 17 | Isolated hyperfibrinolysis | 3.7 | Female | 24 | Menorrhagia, epistaxis, bleeding after tooth extraction and surgery, postpartum bleeding |
| 18 | Isolated hyperfibrinolysis | 4.2 | Female | 12 | Bleeding after surgery and tooth extraction |
| 19 | Isolated hyperfibrinolysis | 4.4 | Female | 10 | Bleeding after surgery |
| 20 | Isolated hyperfibrinolysis | 4.8 | Female | 10 | Postpartum bleeding, bleeding after tooth extraction |
| 21 | Isolated hyperfibrinolysis | 5.9 | Male | 16 | Bleeding after surgery, gastrointestinal bleeding, bleeding after tooth extraction |
| 22 | Isolated hyperfibrinolysis | 6.0 | Female | 12 | Menorrhagia |
| 23 | Isolated hyperfibrinolysis | 6.0 | Female | 16 | Bleeding after surgery, postpartum bleeding |
| 24 | Isolated hyperfibrinolysis | 6.3 | Female | 13 | Bleeding after surgery, postpartum bleeding, bleeding after tooth extraction |
| 25 | Isolated hyperfibrinolysis | 7.2 | Female | 15 | Bleeding after tooth extraction, postpartum bleeding |
| 26 | Isolated hyperfibrinolysis | 8.3 | Female | 8 | Bleeding after tooth extraction |
| 27 | Isolated hyperfibrinolysis | 8.5 | Female | 11 | Menorrhagia, ovarian cyst hemorrhage |
| 28 | Isolated hyperfibrinolysis | 12.1 | Female | 14 | Bleeding after surgery, postpartum bleeding |
| 29 | Isolated hyperfibrinolysis | 14.5 | Male | 8 | Unknown |

**Supplementary Table S5: Individual patient characteristics and main bleeding manifestations for patients with PAI-1 deficiency and isolated hyperfibrinolysis.**
† ISTH-BAT items that scored 3 or 4 points were considered the main bleeding presentations and are listed in order of decreasing score. Items scoring 3 or 4 points based solely on prophylactic use of hemostatic agents were excluded. In patients 1 and 28, who had no items scoring 3 or 4 points, items that scored 2 points were considered the main bleeding presentation. ISTH-BAT = International Society on Thrombosis and Hemostasis bleeding assessment tool; PAI-1 = Plasminogen Activator Inhibitor 1.
